# Rat Lungworm Infection Associated with Central Nervous System Disease — Eight U.S. States, January 2011–January 2017

**DOI:** 10.15585/mmwr.mm6730a4

**Published:** 2018-08-03

**Authors:** Eugene W. Liu, Brian S. Schwartz, Nicholas D. Hysmith, John P. DeVincenzo, Derek T. Larson, Ryan C. Maves, Debra L. Palazzi, Chelsea Meyer, Haidee T. Custodio, Mariejane M. Braza, Roukaya Al Hammoud, Suchitra Rao, Yvonne Qvarnstrom, Michael J. Yabsley, Richard S. Bradbury, Susan P. Montgomery

**Affiliations:** ^1^Epidemic Intelligence Service, CDC; ^2^Division of Parasitic Diseases and Malaria, Center for Global Health, CDC; ^3^Division of Infectious Diseases, University of California, San Francisco, California; ^4^Division of Infectious Diseases, University of Tennessee College of Medicine, Memphis, Tennessee; ^5^Division of Infectious Diseases, Naval Medical Center, San Diego, California; ^6^Infectious Diseases Section, Baylor College of Medicine, Texas Children's Hospital, Houston, Texas; ^7^Department of Neurology, University of Utah, Salt Lake City, Utah; ^8^Pediatric Infectious Diseases, University of South Alabama College of Medicine, Mobile, Alabama; ^9^East Texas Infectious Disease Consultants, Tyler, Texas; ^10^Division of Pediatric Infectious Diseases, The University of Texas Health Science Center at Houston, McGovern Medical School, Houston, Texas; ^11^Division of Pediatric Infectious Diseases and Hospital Medicine, Children's Hospital Colorado and University of Colorado, Aurora, Colorado; ^12^Southeastern Cooperative Wildlife Disease Study, Department of Population Health, College of Veterinary Medicine and the Warnell School of Forestry and Natural Resources, University of Georgia, Athens, Georgia.

Angiostrongyliasis is caused by infection and migration to the brain of larvae of the parasitic nematode *Angiostrongylus cantonensis*, or rat lungworm. Adult *A. cantonensis* reside in the lungs of the definitive wild rodent host, where they produce larvae passed in feces, which are then ingested by snails and slugs (gastropods). Human infection typically occurs when gastropods containing mature larvae are inadvertently ingested by humans. Although human infection often is asymptomatic or involves transient mild symptoms, larval migration to the brain can lead to eosinophilic meningitis, focal neurologic deficits, coma, and death. The majority of cases of human angiostrongyliasis occur in Asia and the Pacific Islands, including Hawaii, but autochthonous and imported cases have been reported in the continental United States (*1*,*2*), underscoring the importance of provider recognition to ensure prompt identification and treatment. The epidemiologic and clinical features of 12 angiostrongyliasis cases in the continental United States were analyzed. These cases were identified through *A. cantonensis* polymerase chain reaction (PCR) testing (*3*) of cerebrospinal fluid (CSF) submitted to CDC from within the continental United States. Six cases were likely a result of autochthonous transmission in the southern United States. All 12 patients had CSF pleocytosis and eosinophilia, consistent with eosinophilic meningitis. Health care providers need to be aware of the possibility of angiostrongyliasis in patients with eosinophilic meningitis, especially in residents in the southern United States or persons who have traveled outside the continental United States and have a history of ingestion of gastropods or contaminated raw vegetables.

Cases of human angiostrongyliasis were identified through review of results of *A. cantonensis* PCR (*3*) testing performed at CDC on CSF specimens from January 2011, when this test became available, through January 2017. A presumptive case was defined as detection of *A. cantonensis* DNA by PCR testing of a clinical CSF specimen submitted to CDC by a diagnostic laboratory located in the continental United States. A confirmed case was a presumptive case with health care provider documentation of clinically compatible disease. Clinical and epidemiologic information was also obtained directly from providers based on review of medical records with patient consent and, when available, from published case reports. Abstracted data included vital signs, clinical signs and symptoms at the time of initial evaluation, hospital course, clinical progression, and laboratory data.

*A. cantonensis* DNA was detected in 34 (49.3%) of 69 persons whose CSF specimens were received and tested by CDC during January 2011–January 2017. Specimens from 17 of these 34 patients were submitted from within the continental United States and, therefore, were considered presumptive angiostrongyliasis cases. In one presumptive case, the patient was determined by the provider to have an alternative diagnosis, and the PCR result (which required >32 cycles to detect DNA) was considered to be a false positive. Among the remaining 16 presumptive cases, the median patient age was 20 years (range = 1–68 years), and 10 were male. The 16 patient specimens were submitted from eight states, including six from California, four from Texas, and one each from Utah, Colorado, Arizona, Alabama, Tennessee, and New York. Eight patients had traveled to areas outside the continental United States (Asia, the Caribbean, or Pacific Islands) during the 12 months preceding initial evaluation, six (four in Texas and one each in Tennessee and Alabama) had no history of travel outside the continental United States, and the travel history was unknown for two patients in California.

Six of 11 patients reported consumption of raw vegetables, three from local gardens (Table 1). Two of 12 patients had consumed raw snails and for two others, family members reported the presence of snails in the environment. Two of nine patients reported consumption of prawns (with one patient specifying the prawns as being cooked), and one of nine patients ate cooked crab. One of 11 patients was reported to have consumed slugs, and one had possible exposure to slugs. Among the six patients who had not traveled outside the continental United States, two had consumed raw vegetables, three had possible exposure to snails or slugs, and one had a history of geophagia.

**TABLE 1:** Exposures reported in 16 patients with presumed angiostrongyliasis with detectable *Angiostrongylus cantonensis* DNA on polymerase chain reaction testing at CDC — continental United States, January 2011–January 2017

Exposure	No. of exposures		
	Yes (%)*	Possible	No
Raw vegetables^†^	6/11 (55)	0	5
Prawns	2/9 (22)	0	7
Snails	2/12 (17)	2	8
Crabs	1/9 (11)	0	8
Slugs	1/11 (9)	1^§^	9
Frogs	0/8 (0)	0	8

* Percentages were calculated using denominators based on availability of complete exposure data.

**^†^** Three patients were known to have consumed vegetables from a local garden.

^§^ This patient was a toddler who was often permitted to crawl in a yard known to contain slugs.

A diagnosis of angiostrongyliasis was confirmed in 13 of the 16 presumptive cases from providers (10 patients), published case reports (two) (*1*,*4*), and personal communications (one). Complete clinical information was available for 12 of these patients (Table 2). The most frequently reported symptoms were subjective fever, generalized weakness, headache, and numbness/tingling. Neurologic exam findings during initial evaluation included cranial nerve deficits (five of 11), nuchal rigidity (four of 12), focal weakness (three of 10), and paresthesias (one of eight). Irritability was noted in three patients, two of whom also had ataxia during the initial evaluation; a separate patient had ataxia at 20 days. During initial evaluation, 10 of 12 patients had peripheral eosinophilia (>600 eosinophils/mm^3^). All 12 patients with CSF microscopy and chemistry results had pleocytosis during initial evaluation; 10 had CSF eosinophilia (≥10% of all leukocytes in CSF or ≥10 eosinophils/mm^3^) on initial evaluation and two on subsequent lumbar puncture. Six of 11 patients also had hypoglycorrhachia (CSF glucose <40 mg/dL) at the time of initial evaluation. Repeat lumbar punctures were performed in eight of 11 patients. On magnetic resonance imaging or computed tomography, eight of 11 patients had brain abnormalities, and five of six had spinal cord abnormalities. Abnormalities were also observed in the optic nerve of two patients. A chest computed tomography scan in one patient had multiple small focal areas of consolidation.

**TABLE 2:** Symptoms, physical exam findings, and laboratory results for 12 patients with angiostrongyliasis with detectable *Angiostrongylus cantonensis* DNA on polymerase chain reaction testing at CDC — continental United States, January 2011–January 2017

Observation/Finding*	Present, No.	Absent, No.	Proportion with symptom/sign present (%)^†^
**Symptom/Sign**			
Subjective fever	8	2	8/10 (80)
Generalized weakness	7	2	7/9 (78)
Headache	6	2	6/8 (75)
Numbness/Tingling	3	3	3/6 (50)
Photophobia	4	5	4/9 (44)
Visual changes	3	4	3/7 (43)
Vomiting	3	6	3/9 (33)
Stiff neck	2	7	2/9 (22)
Rash	2	7	2/9 (22)
Nausea	1	5	1/6 (17)
Phonophobia	1	6	1/7 (14)
Abdominal pain	1	7	1/8 (13)
Itching	1	8	1/9 (11)
Diarrhea	3	NA	NA
Hyperesthesias/diffuse allodynia	2	NA	NA
**Physical exam**			
**Vital signs**			
Fever (temperature ≥100.4°F [≥38.0°C])	3	8	3/11 (27)
Tachycardia (>100 bpm in adults aged ≥16 yrs, age-dependent in persons aged <16 yrs)	1	10	1/11 (9)
Hypoxia (O_2_ saturation <90%)	0	10	0/10 (0)
**Neurologic exam findings**			
Cranial nerve deficits	5	6	5/11 (45)
Nuchal rigidity	4	8	4/12 (33)
Focal weakness	3	7	3/10 (30)
Paresthesias	1	7	1/8 (12)
Loss of consciousness	0	10	0/10 (0)
Irritability	3	NA	NA
Ataxia	2	1^§^	NA
**Laboratory results on initial evaluation**			
**Cerebrospinal fluid**			
Pleocytosis of CSF (≥6 WBC/mm^3^)	12	0	12/12 (100)
CSF eosinophilia (eosinophils ≥10% of all leukocytes in CSF or ≥10 eosinophils/mm^3^)	10	2^¶^	10/12 (83)
Hypoglycorrhachia (CSF glucose <40 mg/dL)	6	5	6/11 (54)
**Complete blood count**			
Peripheral eosinophilia (>600 eosinophils/mm^3^)	8	2	8/10 (80)
Leukocytosis (>11x10^3^ WBC/mm^3^ in persons aged >21 yrs, age-dependent in persons aged ≤21 yrs)	3	9	3/12 (25)

**Abbreviations:** bpm = beats per minute; CSF = cerebrospinal fluid; NA = not available; O_2_ = oxygen; WBC = white blood cells.

*Confirmed by the patient’s health care provider

^†^ Percentages were calculated with different denominators based on availability of complete clinical data.

^§^ This patient developed ataxia 20 days after initial evaluation.

^¶^ These two patients were found to have CSF eosinophilia on repeat lumbar puncture.

Eleven of 12 patients with confirmed cases received systemic steroids, as advised in treatment recommendations (*5*). Seven patients received an antiparasitic (albendazole). Two months after initial evaluation, all 12 patients were alive, 11 had improvement of symptoms, and four had ongoing focal neurologic symptoms (cranial nerve palsies or lower extremity weakness). Only one patient developed seizures (5 months after the initial diagnosis) for which antiepileptics were given.

## Discussion

Among 12 confirmed cases of angiostrongyliasis in the continental United States during January 2011–January 2017, six likely resulted from autochthonous transmission in the southern United States. The possibility of autochthonous transmission is supported by evidence of infection with *A. cantonensis* among intermediate snail hosts and nonhuman vertebrate hosts in the southern United States. Infection has been observed in exotic and native snail species in Florida and Louisiana (*6*,*7*) and in rat species in Louisiana, Florida, and Oklahoma (*6*). Infection with larvae has been documented in other vertebrates including opossums and nine-banded armadillos in Louisiana and Florida (*8*), an American miniature horse in Mississippi, and captive exotic primates in Louisiana, Florida, and Alabama (*7*).

The majority of patients in this series had subjective fever, generalized weakness, headache, and CSF pleocytosis consistent with meningitis. Most also had presence of eosinophils in both peripheral blood and CSF, and hypoglycorrhachia, which is usually associated with bacterial, fungal, or tuberculous meningitis. All 12 patients eventually developed CSF eosinophilia, as did all hospitalized patients during a 2000 outbreak of eosinophilic meningitis caused by *A. cantonensis* among travelers to the Caribbean (*2*). Although no specific treatment for *A. cantonensis* infection currently exists (https://www.cdc.gov/parasites/angiostrongylus/), nearly all patients in this series were treated with systemic steroids, which have been determined to decrease the duration of headaches (*5*), and approximately half of patients were treated with albendazole, for which conflicting evidence of efficacy in treating headache can be found (*9*,*10*). Whether these treatments affected the clinical course for these patients is unclear.

The findings in this report are subject to at least two limitations. First, all cases in this series were identified from specimens tested at CDC, and consequently might not be a comprehensive description of all illnesses caused by *A. cantonensis* in the continental United States during this time. Second, exposures are incompletely reported in some cases, and clinical histories from three presumptive cases could not be obtained.

Health care providers in the United States, especially those in areas in the southern United States where autochthonous cases have been reported, need to be aware of the possibility of angiostrongyliasis in patients with eosinophilic meningitis. Ingestion of gastropods or locally obtained raw vegetables* contaminated with *A. cantonensis* larvae in the southern United States, even in the absence of a travel history, should increase provider suspicion for angiostrongyliasis.

**Summary**

**What is already known about this topic?**

Ingestion of snails or slugs containing *Angiostrongylus cantonensis* larvae can result in angiostrongyliasis, characterized by eosinophilic meningitis. Angiostrongyliasis typically occurs in Asia and the Pacific Islands.

**What is added by this report?**

CDC identified 12 angiostrongyliasis cases in the continental United States occurring from January 2011 through January 2017. Consumption of raw vegetables was reported in the majority of cases (55%). Six were likely autochthonous cases occurring in the southern United States.

**What are the implications for public health practice?**

Health care providers, especially those in the southern United States, need to consider angiostrongyliasis in patients with eosinophilic meningitis, particularly those with a history of ingestion of gastropods or raw vegetables contaminated with larvae.
